# Supplementary material for: All-Optical, Air-Coupled Ultrasonic Detection of Low-Pressure Gas Leaks and Observation of Jet Tones in the MHz Range
Source: Sensors (Basel). 2023 Jun 17;23(12):5665. doi: 10.3390/s23125665 (PMC10304996; doi:10.3390/s23125665)
Supplement: Supplementary file 1 [file sensors-23-05665-s001.zip › sensors-2445952-supplementary.pdf]

Supplemental Information for "All-Optical, Air-Coupled Ultrasonic Detection of Low-Pressure Gas Leaks and Observation of Jet Tones in the MHz Range"

Kyle G. Scheuer [1,*] and Ray G. DeCorby [2,*]

[1] Ultracoustics Technologies Inc., Sherwood Park, AB T8A 3H5, Canada
[2] ECE Department, University of Alberta, 9211-116 St. NW, Edmonton, AB T6G 1H9, Canada
* Correspondence: kyle@ultracoustics.ca (K.G.S.); rdecorby@ualberta.ca (R.G.D.)

As referenced in the main manuscript, needle assemblies were made with three different needle gauges. Figure S1 shows a photograph of all three assemblies disconnected from the gas handling system. Each assembly was built by first sanding down the inside edge of a syringe to roughly match the external diameter of the tubing used before permanently bonding the syringe and tubing using a cyanoacrylate-based glue. The needle was also threaded into the syringe and fixed in place with glue. A brass fitting was installed at the opposite end so that each needle assembly could be directly attached to the needle valve contained in the gas handling system.

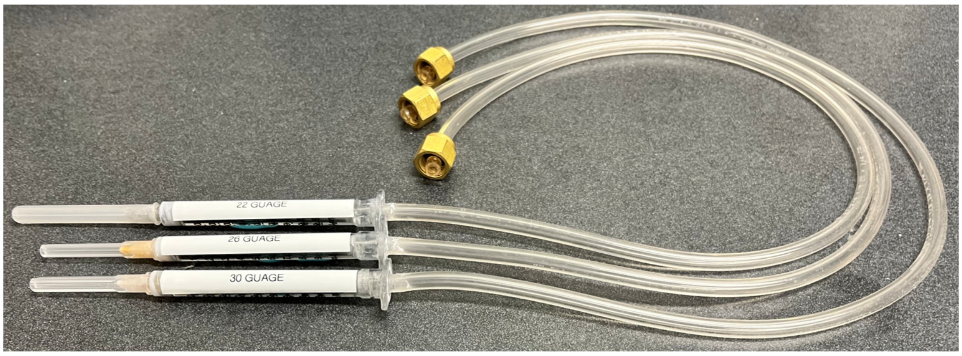

Figure S1. A photograph of the needle assemblies from the main manuscript.

In addition to the full pressure versus frequency characterization for the 30-gauge needle shown in the main text, we also performed the same characterization for the 22- and 26-gauge needles as shown in Fig. S2. We noted that the pressure range where the jet tones occurred was both lower and narrower as the gauge of the needle was decreased.

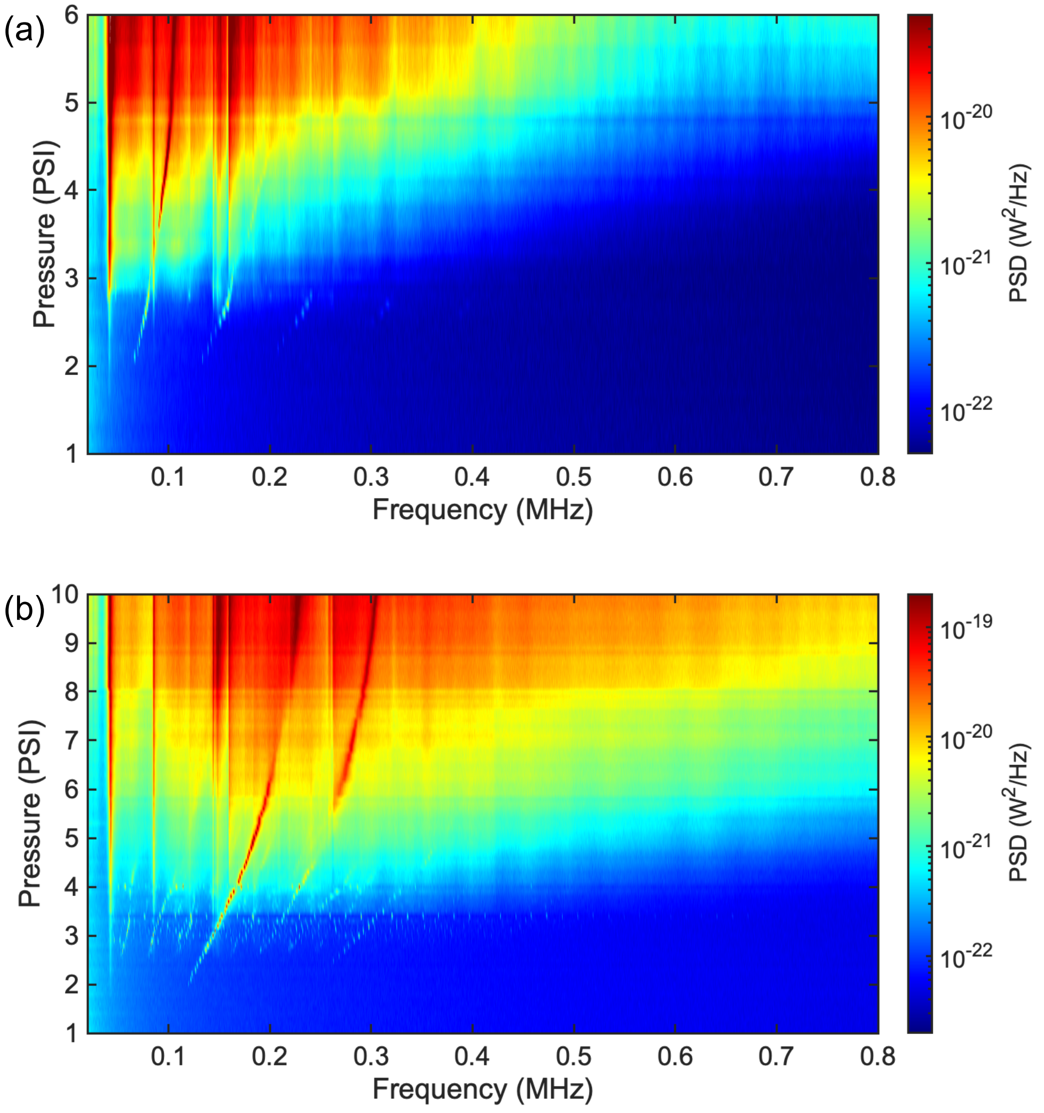

Figure S2. Generation of MHz frequency jet tones with 22- and 26-gauge needles at various pressures. (a) A colormap characterizing the jet tones as a function of pressure for the 22-gauge needle. (b) A colormap characterizing the jet tones as a function of pressure for the 26-gauge needle.

As shown in Fig. 4 of the main text, as well as previous work [1,2], our sensors have resonant features that extend far into the MHz range. One such feature is located near 2.35 MHz and is visible regardless of the pressure. It is possible to account for the impact of these features by taking the ratio of the power spectral density, as shown in Fig. S3. Note that the mechanical resonance inherent to the dome is no longer present, revealing the lineshape of the white noise contribution from the gas flow, which is only interrupted by the non-linear device-related feature near 4.7 MHz. We also note that expression contained in the y-axis defines a quasi-signal-to-noise ratio, which has a maximum value of ~ 28 dB and is > 1 over the range 0 ~ 5 MHz under this set of test conditions.

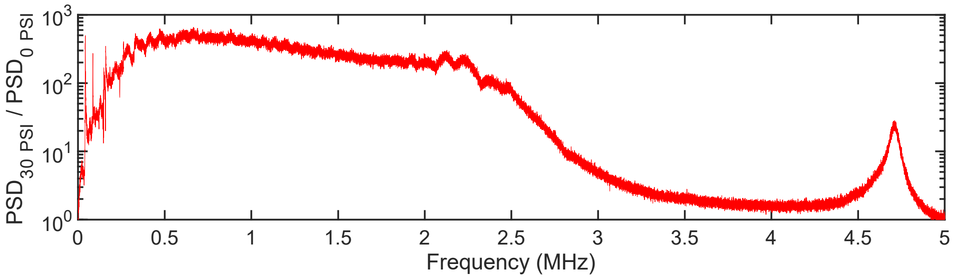

Figure S3. Extracted lineshape for the 30-gauge needle pressurized to 30.0 PSI.

Since we also observed resonances that were static in frequency with pressure, it was also necessary to determine where these tones originated. To do this, we removed the needle assembly and needle valve from the system, leaving only the toggle valve connected to the regulator. The probe was placed at an arbitrary distance from the gas line opening (so that a dominant white noise contribution could be avoided) and the toggle valve was opened fully, allowing the ~ 40 PSI nitrogen to flow freely. Figure S4 shows the frequency content for this case, note the particularly strong features near 42 KHz and 85 KHz that were also present for each needle at low pressures. This represents yet another that our device can be used to detect potential gas leaks.

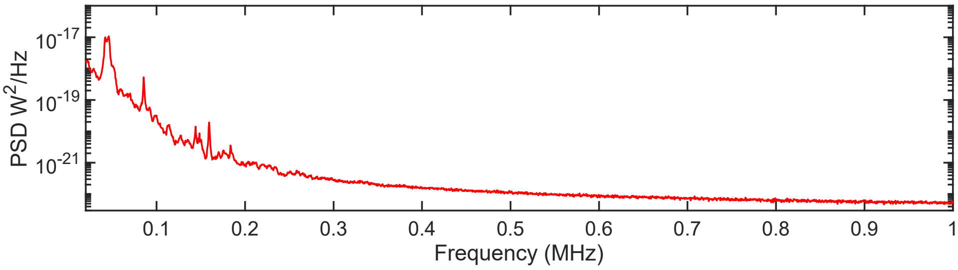

Figure S4. Spectral content inherent to the gas system without a needle assembly in place. This plot is representative of ~ 40 PSI nitrogen flowing out of the gas line.

References

1. Bitarafan, M.H.; Ramp, H.; Allen, T.W.; Potts, C.; Rojas, X.; MacDonald, A.J.R.; Davis, J.P.; DeCorby, R.G. Thermomechanical Characterization of On-Chip Buckled Dome Fabry–Perot Microcavities. *J. Opt. Soc. Am. B* **2015**, *32*, 1214, doi:10.1364/josab.32.001214.
2. Hornig, G.J.; Scheuer, K.G.; Dew, E.B.; Zemp, R.; DeCorby, R.G. Ultrasound Sensing at Thermomechanical Limits with Optomechanical Buckled-Dome Microcavities. *Opt. Express* **2022**, *30*, 33083–33096, doi:10.1364/OE.463588.
